# A Gossip about Pills

**Published:** 1890-04-19

**Authors:** 


### The Doctor's Pulpit.

### A GOSSIP ABOUT PILLS.

Should a preacher, even so secular a preacher as the doctor, ever indulge in gossip? Perhaps we may widen the question and ask if all preachers of every sort may not sometimes descend to that kind of homely and pleasant talk which the word "gossip" so excellently defines? We venture to think they may. What was Charles Lamb but a gossip; and who among English essayists is so well beloved and so well read as he? John Foster talked excellent sense in admirable sentences, but those who read him to-day are certainly not legion. Even A. K. H. B., inconsequent twaddler though he often was, yet numbered his readers by tens of thousands, and solely because his gossip was so uncommonly easy and pleasant. The subject of "Pills," moreover, appears to demand the gossipy style of discussion. But if that be so, says the man of preternatural solemnity, why discuss it at all? Why? Because to nearly all civilised mankind it is one of the burning practical questions of the day.

"What do you think of 'Cockle'"? innocently asks an unsophisticated patient of the dignified and orthodox family physician. "Of Cockle, my dear madam, of Cockle? I never think anything at all of Cockle." But the patient thinks of Cockle, and of Holloway, and Morrison, and Beecham, and all the rest of them. The "Great Pill Question" is, indeed, one of immediate and daily significance. Ought I to take pills? And if I ought, whose pills should they be? Even you, Dr. Orthodoxy, will admit that this is an inquiry which the town man is frequently compelled to make, and to which he is entitled to have an answer.

Puppies do not get their eyes open until they are nine days old. They manage to survive, notwithstanding. But it is plain that the puppy, after he has got his eyes open, is at least more lively, and he is presumably also more happy. He is certainly more successful in effecting everything that goes to constitute the complete duty of puppydom; and there is no doubt that both he who uses them, if he could speak, and we who look on would say that, on the whole, "eyes" are very good things for puppies. But if eyes are good and even necessary for the puppy in the puppy stage, much more are they necessary as development advances and as environment becomes more complex. Do not be alarmed at the big words, amiable reader! They only mean that the dog can kill rats, clear the garden of cats, and enjoy a tussle with his neighbour dog much better with eyes than he could without them. The human baby is born with his eyes open, and from this and other indications that need not be more particularly specified, we infer that both as child and as man, the use of his eyes is to be one of his principal occupations in life.

What has all this to do with pills; above all, with patent pills? Everything. This is an age of knowledge, of open-eyed knowledge. Nobody likes to do anything without first understanding all about it. There are very few Columbuses who venture life and reputation in search of undiscovered countries in these times. Even the youthful plough-boy will tell you that he is too wide awake to "buy a pig in a poke." He thinks it might turn out a guinea pig, or even a lean and hungry tabby. But if a raw farm labourer will not buy a pig in a poke, is it not strange that even a clever man of business will take a pill whose properties he knows nothing at all about? This is the central idea of our brief "gossip," and we invite readers to consider it intelligently. All town people, it must be borne in mind, are more or less familiar with the use and peculiar action of common drugs. It would be very difficult to find in London a fairly cultivated man who does not know something about their physiological effects. He is quite aware, for example, that quinine is a tonic; that mercury and podophyllin act upon the liver; that opium takes away pain; and that rhubarb, aloes, and Epsom salts are aperients. He knows these things quite well, and many other medical facts besides.

Our suggestion is that the man or woman who uses a family pill on his or her own responsibility and without the intervention of a doctor is entitled to know what drugs it is actually composed of, and the exact quantity of each drug it contains. Many substances, most substances, in fact, that possess aperient properties have other properties as well, some of them injurious, and some decidedly dangerous. Mercury, for example, is one of the commonest of all ingredients in what are called "liver pills." A single pill containing a small quantity of mercury has been known to produce salivation, ulceration of the gums, loosening of the teeth, a vile odour of the breath, feverishness, weakness, and a whole series of other symptoms sometimes requiring treatment in bed for a week or two. Though it is rare for a single pill to produce this marked effect, two or three pills taken on successive nights often do it, and particularly in delicate women. We do not hesitate to say that no man or woman should take mercury without a qualified medical man's instructions, unless he has previously proved by repeated personal experience that mercury is quite safe for him. Podophyllin is less dangerous than mercury, but also much more painful in its action, and very debilitating. Even quinine may produce serious symptoms in some persons, and in peculiar bodily conditions. Opium, which is, perhaps, one of the commonest ingredients employed by patent medicine vendors in the preparation of their wares, is one of the most dangerous poisons known.

The contention is that men and women who use patent pills and such other articles not only "buy a pig in a poke," but do so without the justification of necessity. They would not take a chicken from the poulterer, or a sirloin from the butcher, still less a bonnet from the milliner or a coat from the tailor, without previous careful inspection and full knowledge on their own part. Yet it would be much wiser and safer to do any or all these things than to take pills when they are out of health, of whose contents and properties they know nothing at all. It may be laid down as a general principle that no man should do a thing with his eyes shut if he have the chance of doing it with them open. A leap in the dark should never be taken except under the sternest necessity. Does it require any argument to show that this principle is as applicable to pills as it is to politics or to every-day business?

But says the man who prides himself on his common sense and his defiance of professional bugbears—"What do you propose to substitute that shall be as convenient and as efficacious as the patent pill"? Our proposal would be that every man should have his own personal pill, and every family its own family pills, and that they should know fully and accurately what those pills contain. An arrangement of this kind is the easiest thing in the world to bring about. The family or consulting physician will readily write out a prescription for such a pill, and will translate his medical Latin into plain English when so desired. This, we suggest, is a rational and civilized method of procedure. It gets rid at a stroke both of medical mysteries and quack pretences, and at the same time it lets men and women know exactly what they are taking. Of course, no writer, professional or otherwise, can furnish his readers with common sense. But if there be any rational objections against the plan here proposed the writer would like to know what they are. In his judgment any man who walks in darkness when he might walk in light is certainly the opposite of a philosopher.

